# International Circumpolar Surveillance, An Arctic Network for the Surveillance of Infectious Diseases

**DOI:** 10.3201/eid1401.070717

**Published:** 2008-01

**Authors:** Alan J. Parkinson, Michael G. Bruce, Tammy Zulz, the International Circumpolar Surveillance Steering Committee

**Affiliations:** * Centers for Disease Control and Prevention, Anchorage, Alaska, USA

**Keywords:** Arctic, surveillance, infectious diseases, perspective

## Abstract

Peoples of the Arctic and sub-Arctic regions live in social and physical environments that differ substantially from those of their more southern-dwelling counterparts. The cold northern climate keeps people indoors, amplifying the effects of household crowding, smoking, and inadequate ventilation on person-to-person spread of infectious disease. The emergence of antimicrobial drug resistance among bacterial pathogens, the reemergence of tuberculosis, the entrance of HIV into Arctic communities, and the specter of pandemic influenza or the sudden emergence and introduction of new viral pathogens such as severe acute respiratory syndrome are of increasing concern to residents, governments, and public health authorities. The International Circumpolar Surveillance system is a network of hospital, public health agencies, and reference laboratories throughout the Arctic linked together to collect, compare, and share uniform laboratory and epidemiologic data on infectious diseases and assist in the formulation of prevention and control strategies.

## Abstract 2

Hospitals, public health agencies, and reference laboratories work together to detect and control infectious disease in Arctic regions.

Arctic populations have historically endured the debilitating effects of both endemic and epidemic infectious diseases (*1*,*2*). The introduction of antimicrobial drugs and vaccines and the establishment of robust public health systems have greatly reduced illness and deaths caused by infectious diseases in many Arctic countries. Despite these interventions, high rates of invasive diseases caused by bacterial pathogens such as *Streptococcus pneumoniae* (*3*–*5*), *Haemophilus influenzae* (*6*), *Helicobacter pylori* (*7*,*8*), and *Mycobacterium tuberculosis* (*9*–*12*) continue to persist. In addition, the emergence of antimicrobial drug resistance among bacterial pathogens once easily treated with commonly used antibiotics (*10*,*13*–*15*), the entrance of HIV into Arctic communities (*10*,*16*), and the specter of pandemic influenza or the sudden emergence and introduction of new viral pathogens such as severe acute respiratory syndrome (SARS) are of increasing concern to residents, governments, and public health authorities of all Arctic countries.

## Social and Physical Environment

**Figure 1:**
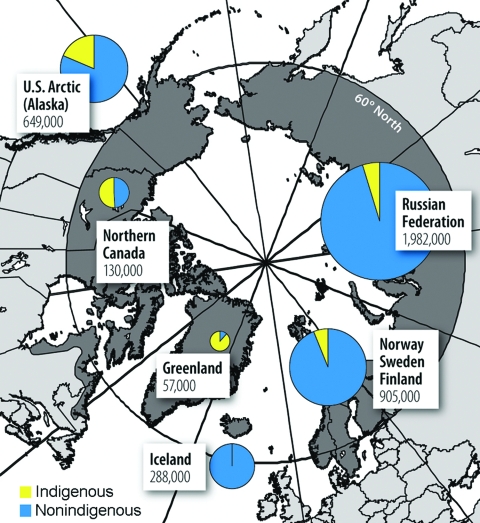
The circumpolar region and nonindigenous and indigenous populations of the Arctic. (Adapted from [*17*].)

Peoples of the Arctic and sub-Arctic regions live in social and physical environments that differ substantially from those of their more southern-dwelling counterparts (*17*). The circumpolar region can be defined as a region that extends north of 60° north latitude, borders the Arctic Ocean, and includes all or northern parts of 8 nations: the United States (Alaska), Canada, Greenland, Iceland, Norway, Finland, Sweden, and the Russian Federation (Figure 1). Climate in the Arctic varies geographically from severe cold in arid uninhabited regions to temperate forests bordering coastal agrarian regions.

Approximately 4 million people live in the Arctic; almost half reside in northern regions of the Russian Federation. The populations of these countries comprise varying proportions of European and indigenous ancestry. The Arctic is the homeland of the Eskimo; the Inuit of Greenland, northern Canada, and northern Alaska; the Yupik of western Alaska and eastern coastal regions of the Russian Far East; and the Aleut of the Aleutian Chain. The interior and western coastal regions of northern Canada and Alaska are the homes of a wide variety of North American Indian, linguistically distinct groups, including the Athabaskan, Eyak, Tlingit, Gwich’in, and Metis. In Alaska the collective terminology for persons of indigenous ancestry is Alaska Native. Although the group is not registered in official statistics, the Saami people inhabit circumpolar regions of Norway, Finland, and Sweden. The Russian census recognizes (from West to East) the Saami, Nenets, Khanty, Sel’kup, Enets, Nganasan, Dolgan, Even, Chukchi, Chuvan, and Eskimo/Inuit-Yupik of the Russian Far East. Arctic populations have certain demographic characteristics that separate them from populations in more southern regions. Birth and mortality rates are higher, and life expectancy is lower; a high proportion of the population is thus of younger age (*1*). In remote regions of the North American Arctic, Greenland, and the northern Russian Federation, many residents live in small, isolated communities that are dependent on hunting and fishing with little or no economic infrastructure. In these remote regions, public health and acute-care systems are often marginal, sometimes poorly supported, and in some cases nonexistent.

The Arctic is well known as a vast source of natural resources such as fish, forests, oil, gas, and metal ores. Exploitation of these resources requires both infrastructure development and improved transportation. However, communities once isolated are now linked by air to larger urban centers, which provides daily access not only to secondary and tertiary healthcare, but also to national and international transportation, tourism, and trade. In Iceland, northern Norway, Finland, Sweden, and the more densely populated regions of the North American Arctic, Greenland, and the northern Russia Federation, a more affluent economic mixture has emerged because of light industry; oil, gas, and mineral development; transportation and agriculture; and more sophisticated systems of healthcare and public health.

## Inadequate Housing/Crowded Living Conditions

In smaller isolated communities, inadequate housing is an important determinant of infectious diseases. The cold northern climate keeps persons indoors, which amplifies the effects of household crowding, smoking, and inadequate ventilation. Crowded living conditions increase person-to-person spread of infectious diseases and favor the transmission of respiratory diseases (*18*–*20*), tuberculosis (*12*), gastrointestinal diseases (*21*), and skin infections (*22*). In many smaller isolated communities, inadequate sewage disposal systems and water supplies pose a substantial risk to health, resulting in periodic epidemics of diseases transmitted by the fecal-oral route (*21*,*23*).

## Overuse of Antimicrobial Drugs

Empiric use, and possible overuse, of antimicrobial agents in remote Arctic regions has contributed to the emergence of bacterial strains now resistant to commonly used antibiotics. In the northern regions of the Russian Federation, underfunding of tuberculosis treatment programs have resulted in an unpredictable supply of antibiotics, which contributes to poor adherence and emergence of multidrug-resistant tuberculosis (*10*). In remote Alaskan villages, lack of ready access to laboratory confirmation of bacterial pathogens may contribute to overuse of antimicrobial agents. In addition, the presence of antimicrobial drug–resistant bacterial clones has led to an increase in infections with multidrug-resistant *S. pneumoniae* (*24*,*25*), methicillin-resistant *Staphylococcus aureus* (*13*), and clarthromycin- and metronidazole-resistant *H. pylori* (*14*).

## Role of Surveillance

As in other parts of the world, a key component of prevention and control of infectious diseases in Arctic regions is surveillance. Effective surveillance can facilitate timely control of outbreaks, inform public health officials’ decisions on resource allocation, and provide data to adjust prevention and control strategies to maximize their effects. For example, population-based surveillance for invasive *H. influenzae* type b (Hib) disease in the US Arctic demonstrated prevaccine incidence rates of invasive disease of 601 cases and 129 cases per 100,000/population in Alaska Native and non-Native children <5 years of age, respectively (*26*). Immunization programs that use the Hib conjugate vaccine were implemented in the US Arctic in 1991 and resulted in a >10-fold decline in Hib cases in Alaska Native children. However, in May of 1996, continued surveillance detected 4 cases of invasive Hib in children <2 years, and duringf the next 12 months 10 cases occurred. Most cases were in infants who received 1–2 doses of Hib vaccine after a statewide change in 1996 to a vaccine that was less immunogenic after the first dose (*27*,*28*). This experience demonstrated the need for continued surveillance after the implementation of a successful vaccine program and the shortcomings of generalizing data from other countries or regions to develop public health policy in the Arctic.

Similarly, surveillance for invasive disease caused by *S. pneumoniae*, established in the US Arctic in 1986, showed that Alaska Natives had the highest reported average overall rate for invasive pneumococcal disease in the world (62 cases/100,000 population), which was 4× higher than the rate for non-Natives (16 cases/100,000) (*5*). Among Alaska Native children <2, the rate was 450 cases/100,000 versus 129 cases/100,000 among non-Native children. This surveillance system first detected decreased susceptibility to penicillin in the mid-1980s. Although these isolates were only moderately resistant to penicillin, resistance to multiple antimicrobial drugs was also found by 1989. Isolates fully resistant to penicillin detected in 1993 were first recovered from patients living in urban points of entry to the US Arctic and were indistinguishable from multidrug-resistant strains circumnavigating the globe (*24*,*25*). In the US Arctic, the proportion of pneumococcal isolates fully resistant to penicillin increased from 0% in 1991 to 6.1% in 1998, and the proportion of isolates that were resistant to >2 classes of antimicrobial agents increased from 4.7% in 1991 to 17.7% in 1998. In the US Arctic, the 23-valent pneumococcal polysaccharide vaccine is recommended for all persons >55 years of age; however, this vaccine remains underused. In 2001, the heptavalent pneumococcal conjugate vaccine (PCV7) was introduced to the childhood vaccination schedule, and by 2003, vaccine-type invasive pneumococcal disease rates had declined by 91% among Alaska Native children <2 years of age and by 80% among non-Native children <2 years of age. A 40% reduction of invasive pneumococcal disease in adults of all ethnicities suggests an indirect impact or herd effect of this vaccine in nonvaccinated persons. In addition, the use of PCV7 in this population has reduced the proportion of invasive disease caused by isolates resistant to penicillin, erythromycin, and cotrimoxazole (*29*).

These examples demonstrate the feasibility of conducting population-based surveillance to monitor the effects of implemented vaccination programs in reducing the extent of invasive disease caused by 2 common bacterial pathogens in an Arctic region. Population-based surveillance of diseases of concern, including invasive bacterial diseases, is conducted by public health agencies in Canada, Greenland, Iceland, Norway, Finland, and Sweden. Linkage of these surveillance systems would create the beginnings of a circumpolar network of hospitals, public health agencies, and reference laboratories throughout the Arctic to collect, compare, and share uniform laboratory and epidemiologic data on infectious diseases of concern, and assist in the formulation of prevention and control strategies.

## International Circumpolar Surveillance

In 1998, the Arctic Investigations Program (AIP) of the Centers for Disease Control and Prevention, together with Health Canada’s Bureau of Infectious Disease Laboratories Centres for Disease Control, now the Public Health Agency of Canada’s Center for Infectious Disease Prevention and Control, proposed the establishment of an International Circumpolar Surveillance (ICS) system for the detection of infectious diseases of concern in the Arctic (*30*). The initial priority for ICS was the invasive bacterial diseases caused *S. pneumoniae*, *H. influenzae*, *Neisseria meningitidis*, and groups A and B streptococci.

ICS capitalizes on existing national infectious disease surveillance systems and existing long-standing circumpolar collaborative relationships forged through the Arctic Council (www.arctic-council.org) and the International Union for Circumpolar Health (www.iuch.org). The Arctic Council is a ministerial forum promoting cooperation and coordination between Arctic nations on common Arctic concerns and provides a unique opportunity to partner with Arctic nation ministries of health, nongovernmental organizations, and indigenous peoples’ organizations to address health concerns of circumpolar communities. The International Union for Circumpolar Health is a nongovernmental organization comprising the memberships of 5 circumpolar health organizations that promote circumpolar cooperation on Arctic human health.

**Figure 2:**
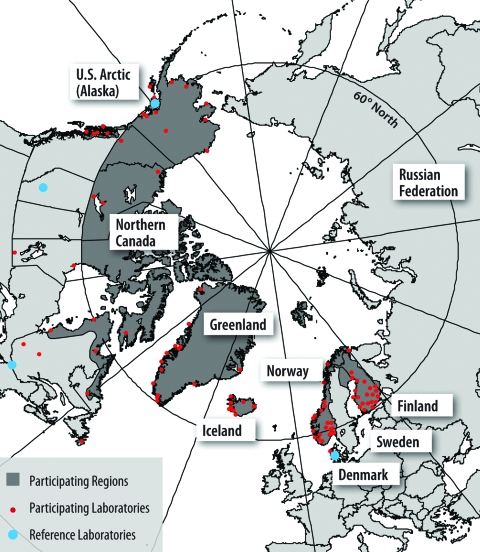
The International Circumpolar Surveillance system participating regions (dark gray), laboratories (small dots), and reference laboratories (large dots).

In 1999, a pilot surveillance system was established to monitor reported cases of invasive pneumococcal disease from 23 clinical laboratories in Alaska and 14 clinical laboratories in the northern Canadian Arctic above 60° north latitude, including the Yukon and Northwest Territories, Nunavut, northern Quebec, and Labrador (Figure 2).

Pneumococcal isolates from patients identified with invasive disease were forwarded to reference laboratories in the US Arctic (AIP) and northern Canada at the National Center for Streptococcus (NCS), Edmonton, Alberta, or Laboratoire de Sante Publique, Quebec (LSPQ), respectively. Identified cases were also reported to local public health personnel, who reviewed and provided clinical, demographic, and vaccination history. Case and culture information was forwarded to the ICS coordinator at AIP for analysis, report generation, and dissemination. In 2000, Greenland joined ICS. Pneumococcal isolates from patients with invasive disease were forwarded from 15 district hospitals first to the Dronning Ingrids Hospital laboratory in Nuuk, Greenland, and then to the Staten Serum Institut (SSI), Copenhagen, Denmark, for serotyping and susceptibility testing. Iceland, Norway, and Finland joined ICS in 2001, reporting national pneumococcal disease surveillance and laboratory information to ICS annually.

Surveillance of other invasive bacterial diseases (*H. influenzae*, *N. meningitidis*, groups A and B streptococci) in the US Arctic, northern Canada, and Greenland was added to ICS in 2000. The northern region of Sweden, Norrbotten, joined ICS in 2003, reporting invasive diseases caused by *S. pneumoniae*, *H. influenzae*, *N. meningitidis*, and groups A and B streptococci.

An ICS quality control exchange program was instituted in 1999 among the 3 reference laboratories (AIP, NCS, LSPQ) for serotyping and antimicrobial susceptibility testing of *S. pneumoniae* (*31*). The program was extended to SSI in 2004. In 2005, an ICS quality control exchange program for serotyping of isolates of *H. influenzae* and *N. meningitidis* was implemented by the National Microbiology Laboratory, Public Health Agency of Canada, in Winnipeg, Manitoba.

The priorities and overall direction of ICS are governed by a steering committee consisting of 2 representatives from each participating country; representation from World Health Organization European regional office, Copenhagen; the Indigenous Peoples Secretariat; and Russian Association of Indigenous Peoples of the North. Other infectious diseases of concern identified by the steering committee , and therefore eligible for circumpolar surveillance, include hepatitis B, tuberculosis, HIV/AIDS, and acute respiratory virus diseases such as respiratory syncytial virus infections in infants. Surveillance of invasive bacterial diseases is coordinated by a subcommittee, the Invasive Bacterial Disease Working Group. As anticipated surveillance for other ICS priority diseases is implemented, similar coordinating working groups led by other partner countries will be established.

Almost half of the circumpolar region consists of northern regions of the Russian Federation, which to its west borders the Arctic regions of Norway and Finland, and to its east is within 2 miles of the US Arctic. The Russian Federation’s communicable disease control systems evolved separately from western public health systems and consist of relatively large federal, regional, disease-specific, sector-specific (prisons, military), and largely clinical case-based reporting systems (*10*). These differences, together with the relative isolation of northern and far-eastern regions of the Russian Federation, the language barrier, and absence of information exchange, have led to some difficulties in establishing cross-border cooperation in infectious disease prevention and control. However, because of a sharp rise in the 1990s in the incidence of communicable diseases such as HIV, sexually transmitted diseases, and tuberculosis (including multidrug-resistant tuberculosis) in the Baltic and Barents Sea regions, several initiatives are now aimed at improving cooperation in infectious disease prevention and control between countries of northwestern Europe and adjacent regions of the Russian Federation. For example, in 1999 the Norwegian Institute of Public Health, together with other Nordic state epidemiologists, established a program to strengthen infectious disease control in the Barents and Baltic Sea states and has since established a framework for communicable disease surveillance communication and training in northern Europe (www.epinorth.org). These activities provide a model for expanding cooperation and developing partnerships for the exchange of infectious disease surveillance information in other northern and far-eastern regions of the Russian Federation.

## Impact of ICS

The ability of ICS to collect and share standardized, uniform laboratory and epidemiologic data on infectious diseases of concern in Arctic countries has already proved valuable in the formulation of prevention and control strategies in regions with small but at-risk Arctic populations. In 2000, ICS data were used to identify an outbreak of *S*. *pneumoniae* serotype 1 invasive disease that occurred among young adults in 2 northern regions of Canada (*32*,*33*). This investigation, together with data from Alaska that indicated that 78%–84% of invasive pneumococcal disease among children <2 years of age could be prevented by using PCV7, resulted in the implementation of vaccine programs in 3 northern regions of Canada using both the 23-valent polysaccharide vaccine and PCV7 in 2002. The introduction of PCV7 in the US Arctic (2001) and northern Canada (2002) has resulted in a rapid decrease in the proportion of isolates resistant to penicillin and other antimicrobial drugs, compared with the situation in countries not using the vaccine (Iceland and Finland) (*34*). Continued surveillance of invasive pneumococcal disease by ICS in these regions will monitor the impact and effectiveness of vaccine programs for preventing invasive pneumococcal disease and antimicrobial drug–resistant infections in these high-risk populations (*34*).

The reemergence of invasive disease caused by Hib in the US Arctic in 1996 following a change in conjugate vaccine type emphasized the need for continued surveillance to monitor vaccine impact, as well as to detect the potential emergence of disease caused by nonvaccine serotypes. Non–Hib (serotypes a, c, d, e, f) is uncommon as a cause of invasive disease in children; however, with the decline in Hib disease, the importance of infections caused by other nonvaccine serotypes has increased. In a 6-month period of 2003, 5 cases of *H. influenzae* type a (Hia) were detected in 3 infants in 1 remote region of the US Arctic (*35*). Between 2000 and 2004, 72 cases of serotype-confirmed *H. influenzae* infection were detected by ICS in Alaska and Canada (*36*). Of these, 34 (47%) were Hia, and 22 (65%) occurred in aboriginal people with a median age of 1.1 years. Hia is now the most common *H. influenzae* serotype seen in the North American Arctic, with the highest rates among indigenous children. Further research is needed to determine sequelae, risk factors, outbreak potential, and the utility of chemoprophylaxis for this disease.

## Arctic Change and Infectious Diseases Surveillance

A common concern among peoples of the Arctic is the rapid pace of economic change and modernization occurring in many communities, which will bring new challenges to the health and well-being of Arctic residents (*19*,*37*). The increasing national and international travel by Arctic residents and increasing access to remote communities by national and international seasonal workforce and tourists have greatly increased the risk of importing infectious diseases to remote communities.

Climate change is also predicted to have major effects within the Arctic (*38*). The average Arctic temperature has risen at almost twice the rate of that in the rest of the world in the last 2 decades and could cause changes in the incidence and geographic distribution of infectious diseases already present in Arctic regions (*39*). For example, an outbreak of *Vibrio parahemolyticus*–related gastroenteritis was reported in July 2004 among cruise ship passengers that consumed raw, farmed oysters in the Prince William Sound area of Alaska (≈60° north latitude), >1,000 km further north than previous reported outbreaks. The July-August water temperature of the oyster farm had increased 0.21°C per year since 1997; 2004 was the first summer on record that the mean water temperature exceeded 15°C, the threshold temperature for the harvest of implicated oysters, which suggests that the ocean warming trend was responsible for this outbreak (*40*). Similarly, higher ambient temperatures in the Arctic may result in an increase in other temperature-sensitive foodborne diseases and influence the incidence of zoonotic infectious diseases by changing the populations and range of animal hosts and insect vectors. The melting of the permafrost together with an increase in extreme weather events such as flooding may result in damage to water and waste disposal systems, which may in turn increase community outbreaks of foodborne and water-borne infections. Temperature and humidity markedly influence the distribution, density, and biting behavior of many arthropod vectors, which again may influence the incidence and northern range of many vector-borne diseases (*39*). These examples emphasize the need for an established surveillance network in Arctic regions for monitoring emerging climate-sensitive infectious diseases.

## Future Directions

ICS provides a model for international surveillance of infectious diseases and collaboration between clinical hospital and public health references laboratories and public health centers and institutes. The system currently provides standardized laboratory and epidemiologic data on invasive bacterial diseases that are comparable across borders and can be used to evaluate intervention strategies. However, the system also provides an infrastructure that can be used to monitor and respond to other emerging infectious disease threats. Tuberculosis presents a continuing challenge to the public health communities of the US Arctic, northern Canada, Greenland, and the Russian Federation. The establishment of an ICS tuberculosis working group would enhance ongoing efforts to reduce the rates of disease in these regions by sharing knowledge, methods, and surveillance data. Because more than half of the circumpolar region is contained within the Russian Federation, efforts should be made to engage and develop partnerships with public health authorities in these regions to learn more about infectious diseases of concern, systems of surveillance, and interests in sharing infectious disease surveillance information.

## References

[1] Bjerregaard P, Young TK, Dewailly E, Ebbesson SO. Indigenous health in the Arctic: an overview of the circumpolar Inuit population. Scand J Public Health. 2004;32:390–5. 10.1080/14034940410028398 15513673

[2] Butler JC, Parkinson AJ, Funk E, Beller M, Hayes G, Hughes JM. Emerging infectious diseases in Alaska and the Arctic: a review and a strategy for the 21st century. Alaska Med. 1999;41:35–43. 10434444

[3] Christiansen J, Poulsen P, Ladefoged K. Invasive pneumococcal disease in Greenland. Scand J Infect Dis. 2004;36:325–9. 10.1080/00365540410020460 15287375

[4] Public Health Agency of Canada. Invasive pneumococcal infection in first Nations children in northern Alberta. Can Commun Dis Rep. 2002;28:165–72. 12402519

[5] Davidson M, Parkinson AJ, Bulkow LR, Fitzgerald MA, Peters HV, Parks DJ. The epidemiology of invasive pneumococcal disease in Alaska: 1986–1990 ethnic differences and opportunities. J Infect Dis. 1994;170:368–76. 8035023 10.1093/infdis/170.2.368

[6] Singleton R, Hammitt L, Hennessy T, Bulkow LR, DeByle C, Parkinson A, The Alaska *Haemophilus influenzae* type b experience: lessons in controlling a vaccine-preventable disease. Pediatrics. 2006;118:e21–9. 10.1542/peds.2006-0287 16882783

[7] Parkinson AJ, Gold BD, Bulkow L, Wainwright RB, Swaminathan B, Khanna B, High prevalence of *Helicobacter pylori* in the Alaska Native population and association with low serum ferritin levels in young adults. Clin Diagn Lab Immunol. 2000;7:885–8. 10.1128/CDLI.7.6.885-888.2000 11063492 PMC95979

[8] Bruce MG, Bruden DL, McMahon BJ, Hennessy TW, Reasonover A, Morris J, Alaska sentinel surveillance for antimicrobial resistance in *Helicobacter pylori* isolates from Alaska Native persons, 1999–2003. Helicobacter. 2006;11:581–8. 10.1111/j.1523-5378.2006.00462.x 17083381

[9] Soborg C, Soborg B, Pouelsen S, Pallisgaard G, Thybo S, Bauer J. Doubling of tuberculosis incidence in Greenland over an 8-year period (1990–1997). Int J Tuberc Lung Dis. 2001;5:257–65. 11326825

[10] Netesov SV, Conrad LJ. Emerging infectious diseases in Russia 1990–1999. Emerg Infect Dis. 2001;7:1–5. 11266288 10.3201/eid0701.010101 PMC2631677

[11] Nguyen D, Proulx JF, Westley J, Thibert L, Dery S, Behr MA. Tuberculosis in the Inuit community of Quebec, Canada. Am J Respir Crit Care Med. 2003;168:1353–7. 10.1164/rccm.200307-910OC 14500266

[12] Gessner BD, Weiss NS, Nolan CM. Risk factors for pediatric tuberculosis infection and disease after household exposure to adult index cases in Alaska. J Pediatr. 1998;132:509–13. 10.1016/S0022-3476(98)70029-0 9544910

[13] Baggett HC, Hennessy TW, Leman R, Hamlin C, Bruden D, Reasonover A. An outbreak of community-onset methicillin resistant *Staphylococcus aureus* skin infections in southwestern Alaska. Infect Control Hosp Epidemiol. 2003;24:397–402. 10.1086/502221 12828314

[14] McMahon BJ, Hennessy TW, Bensler M, Bruden D, Parkinson AJ, Morris JM, The relationship among previous antibiotic use, antimicrobial resistance and treatment outcomes for *Helicobacter pylori* infections. Ann Intern Med. 2003;139:463–9. 13679322 10.7326/0003-4819-139-6-200309160-00008

[15] Rudolph KM, Parkinson AJ, Reasonover AL, Bulkow LR, Parks DJ, Butler JC. Serotype distribution and antimicrobial resistance patterns of invasive isolates of *Streptococcus pneumoniae*: Alaska 1991–1998. J Infect Dis. 2000;182:490–6. 10.1086/315716 10915080

[16] Proceedings of the circumpolar meeting on AIDS prevention. Arctic Med. 1990 (Suppl 3);49:1–38.

[17] Einarsson N, Nymand J, Nilsson OR, eds. Arctic human development report. Akureyi: Steffansson Arctic Institute; 2004.

[18] Bulkow LR, Singleton RJ, Karron RA, Harrisson LH; Alaska RSV Study Group. Risk factors for severe respiratory syncytial virus infection among Alaska Native children. Pediatrics. 2002;109:210–6. 10.1542/peds.109.2.210 11826197

[19] Van Caeseele P, Macaulay A, Orr P, Aoki F, Martin B. Rapid pharmacotherapeutic intervention for an influenza A outbreak in the Canadian Arctic: lessons from Sanikiluaq experience. Int J Circumpolar Health. 2001;60:640–8. 11768446

[20] Karron RA, Singleton RJ, Bulkow L, Parkinson AJ, Kruse D, DeSmet I. Severe respiratory syncytial virus disease in Alaska Native children. J Infect Dis. 1999;180:41–9. 10.1086/314841 10353859

[21] Orr P, Lorencz B, Brown R, An outbreak of diarrhea due to verotoxin-producing *Esherichia coli* in the Canadian Northwest Territories. Scand J Infect Dis. 1994;26:675–84. 10.3109/00365549409008635 7747090

[22] Baggett HC, Hennessy TW, Rudolph K, Bruden D, Reasonover A, Parkinson AJ. Community-onset methicillin-resistant *Staphylococcus aureus*, associated with antibiotic use and cytotoxin Panton-Valentine leukocidin during a furunculosis outbreak in rural Alaska. J Infect Dis. 2004;189:1565–73. 10.1086/383247 15116291

[23] Peach D, McMahon BJ, Bulkow L, Funk B, Harpez R, Margolis HS. Impact of recurrent epidemics of hepatitis A virus infection on population immunity levels: Bristol Bay, Alaska. J Infect Dis. 2002;186:1081–5. 10.1086/343815 12355357

[24] Rudolph KM, Crain MJ, Parkinson AJ, Roberts MC. Characterization of a multi-resistant clone of invasive *Streptococcus pneumoniae* serotype 6B in Alaska using pulsed-field gel electrophoresis and PsPa typing. J Infect Dis. 1999;180:1577–83. 10.1086/315062 10515818

[25] Rudolph KM, Parkinson AJ, Reasonover AL, Bulkow LR, Parks DJ, Butler JC. Serotype distribution and antimicrobial resistance patterns of invasive isolates of *Streptococcus pneumoniae*: Alaska 1991–1998. J Infect Dis. 2000;182:490–6. 10.1086/315716 10915080

[26] Ward JI, Lum MKW, Silimperi DR, Bender TR. Invasive *Haemophilus influenzae* type b disease in Alaska; background epidemiology for a vaccine efficacy trial. J Infect Dis. 1986;153:17–26. 3484505 10.1093/infdis/153.1.17

[27] Galil K, Singleton RS, Levine OS, Fitzgerald MA, Bulkow L, Perkins B, Reemergence of invasive *Haemophilus influenzae* type b disease in a well-vaccinated population in remote Alaska. J Infect Dis. 1999;179:101–6. 10.1086/314569 9841828

[28] Singleton R, Bulkow LR, Levine OS, Parkinson AJ. Experience with the prevention of invasive *Haemophilus influenzae* type b disease by vaccination in Alaska: the impact of persistent oropharyngeal carriage. J Pediatr. 2000;137:313–20. 10.1067/mpd.2000.107843 10969253

[29] Hennessy TW, Singleton RJ, Bulkow LR, Bruden DL, Hurlburt DA, Parks D, Impact of heptavalent pneumococcal vaccine on invasive disease, antimicrobial resistance and colonization in Alaska Natives: progress towards elimination of a health disparity. Vaccine. 2005;23:5464–73. 10.1016/j.vaccine.2005.08.100 16188350

[30] Parkinson AJ, Bell A, Butler JC. International circumpolar surveillance of infectious diseases: monitoring community health in the Arctic. Int J Circumpolar Health. 1999;58:222–5. 10615826

[31] Parkinson AJ, Lovgren M, Jette L, Reasonover A. International Inter-Laboratory Quality Control Program for Circumpolar Surveillance of *Streptococcus pneumoniae.* [Abstract P1576]. Clin Microbiol Infect. 2003;9(Suppl 1):386. 10.1128/JCM.01238-10 PMC3020485 21048017

[32] Proulx JF, Dery S, Jette LP, Ismael J, Libman M, De Wals P. Pneumonia epidemic caused by a virulent strain of *Streptococcus pneumoniae* serotype 1 in Nunavik, Quebec. Can Commun Dis Rep. 2002;28:129–31. 12387098

[33] Macey JF, Roberts A, Lior L, Tam TW, Van Caeseele P. Outbreak of community acquired pneumonia in Nunavut, October and November 2000. Can Commun Dis Rep. 2002;28:131–8. 12387099

[34] Bruce MG, Deeks SL, Zulz T, Bruden D, Navarro C, Lovgren M, International Circumpolar Surveillance system for population-based surveillance of invasive pneumococcal disease, 1999–2005. Emerg Infect Dis. 2008;14:25–33. 10.3201/eid1401.071315 18258073 PMC2600171

[35] Hammitt LL, Block S, Hennessy TW, Debyle C, Peters H, Parkinson A, Outbreak of invasive *Haemophilus influenzae* serotype a disease. Pediatr Infect Dis J. 2005;24:453–6. 10.1097/01.inf.0000160954.90881.29 15876947

[36] Bruce MG, Deeks Sl, Zulz T, Navarro C, Palacios C, Case C, Epidemiology of *Haemophilus influenzae* serotype a, North American Arctic, 2000–2005. Emerg Infect Dis. 2008;14:48–56. 10.3201/eid1401.070822 18258076 PMC2600153

[37] Uyeki TM, Zane SB, Bodnar UR, Fielding KL, Buxton JA, Miller JM, Large summertime influenza A outbreak among tourists in Alaska and Yukon Territory. Clin Infect Dis J. 2003;36:1095–102. 10.1086/374053 12715302

[38] Arctic Council. Arctic climate impact assessment scientific report. Cambridge: Cambridge University Press; 2005. p. 863–906.

[39] Parkinson AJ, Butler JC. Potential impact of climate change on infectious diseases in the Arctic. Int J Circumpolar Health. 2005;64:475–86. 10.3402/ijch.v64i5.18029 16440610

[40] McLaughlin JB, Depoala A, Bopp CA, Martinek KA, Napiolilli NP, Allison CG, Emergence of *Vibro parahaemolyticus* gastroenteritis associated with consumption of Alaskan oysters and its global implications. N Engl J Med. 2005;353:1463–70. 10.1056/NEJMoa051594 16207848

